# Receiving a gift and feeling robbed: a phenomenological study on parents’ experiences of Brief Admissions for teenagers who self-harm at risk for suicide

**DOI:** 10.1186/s13034-023-00675-y

**Published:** 2023-11-08

**Authors:** Reid Lantto, Rose-Marie Lindkvist, Tomas Jungert, Sofie Westling, Kajsa Landgren

**Affiliations:** 1 Department of Clinical Sciences Malmö, Psychiatry, Lund University, Lund, Sweden; 2 Department of Psychology, Lund University, Lund, Sweden

**Keywords:** Phenomenology, Qualitative research, Self-harm, Suicidal ideation, Brief Admission, Self-admission, Self-referral, Adolescents, Parents, Prevention

## Abstract

**Background:**

Brief Admission by self-referral is a preventive intervention here intended for individuals who recurrently self-harm and have a history of contact with emergency psychiatric services. Individuals with access to Brief Admission are empowered to self-admit to inpatient care for up to three days per stay and are encouraged to do so *before* experiencing crisis. Brief Admission was implemented relatively recently in child and adolescent psychiatric settings in Sweden. The purpose of this study was to phenomenologically explore the lived experience of parents whose teenagers, who recurrently self-harm and experience suicidal thoughts, use Brief Admissions.

**Methods:**

This is a qualitative study using phenomenological psychological analysis. We interviewed 17 parents who had experienced their teenagers using Brief Admissions. The interviews were recorded and transcribed verbatim and analyzed to arrive at the essential meaning structure of the phenomenon of Brief Admissions for the parent.

**Results:**

We identified two essential meaning structures of the parent’s experience of their teenager’s use of Brief Admissions: being gifted relief and hope or being robbed of everything you believed in. The experience of Brief Admissions as a gift was structured by the following constituents: ‘a sense of safety and containment’, ‘liberation from a hostage situation’, ‘a return to wellbeing’, and ‘catalysts for relational shifts’. In contrast, the constituents of the experience of being robbed included ‘a tug of war for control’, ‘an unworthy wasteland’, ‘abandonment and collapse of authority’, and ‘no sense of purpose and plan’.

**Conclusions:**

Brief Admissions may come across as challenging, futile and painful in the life of the parent, yet they may also support a process of recovery and healthy development for the entire family. To realize the full potential of the intervention, mental health professionals providing Brief Admission must be mindful of the challenges the parent may face as their teenager starts self-admitting, tactfully and sensitively preparing the parent for a new parental role.

**Supplementary Information:**

The online version contains supplementary material available at 10.1186/s13034-023-00675-y.

## Introduction

Self-harm and suicidal thoughts and behaviors are associated with great suffering, often extending to the surrounding family. Over the past 15 years, research on child and adolescent self-harm and suicidal thoughts and behaviors has increasingly attended to parental perspectives, not only in terms of the parental role in the child’s trajectory, but by considering parents’ experiences, perspectives and needs in their own right (Curtis et al. 2018; Krysinska et al. 2020; Zhao et al. 2022; Weissinger et al. 2023).

Frequently reported in previous literature, parents struggle with meeting their own and other family members’ needs when a child self-harms (Arbuthnott & Lewis 2015), and may experience guilt, shame, doubts, and fears regarding their response to self-harm and their parenting at large (Arbuthnott & Lewis 2015; Curtis et al. 2018; Krysinska et al. 2020). They may even feel that the child’s self-harm is the parent’s fault (McDonald et al. 2007). Consequently, parents may become excessively controlling of, or conversely fearing to impose boundaries for, their child (Curtis et al. 2018). Parent and child may not be able to communicate about self-harm, and the parent may instead learn to infer things about their child’s emotional states from the home environment, becoming anxiously preoccupied with looking for signs of self-harm (Steggals et al. 2020). Further, parents report financial stress as a result of being away from work, increased conflicts within the family, strain in romantic relationships and withdrawal from the extended family and friends, as well as personal physical and mental health problems experienced as linked to their child’s self-harm (Townsend et al. 2021).

Similar findings of guilt, shame and inadequacy, changes in parenting style, strains in other family relationships and strains related to work-life, are echoed in research on parents’ experiences of their child’s suicidal thoughts and behaviors (Buus et al. 2014; Weissinger et al. 2023). Parents are in a constant state of fear and alarm in relation to their teenagers’ suicidal behaviors (Buus et al. 2014; Weissinger et al. 2023). The responsibility for their child’s life is a heavy burden to carry and parents may feel alone and isolated in the world; yet they may have a hard time identifying and focusing on their own experiences and feelings, as they are so absorbed by their suicidal child’s experiences (Weissinger et al. 2023). The child’s suicidal thoughts and behaviors may take a toll on the parent’s identity (Daly 2005; Juel et al. 2023). The parent may also experience a sense of loss, of hope, peace, and the life and child they used to know, and find themselves in a prolonged process of grief (Daly 2005).

In terms of parents’ relation to the healthcare system, psychiatric inpatient care may feel like a prison, holding their child captive (Weissinger et al. 2023). Indeed, even the parent may feel part of a hostage drama, with a sense of being trapped as they lose faith in the healthcare system (Lindgren et al. 2010). Parents often feel that the healthcare their child receives is limited, untimely, fragmented, and inadequate, and they feel excluded from the child’s care interventions and planning. They also may feel like they do not understand enough about their child’s situation and would like to receive psychoeducation and guidance on how best to support their child (Simes et al. 2022; Zhao et al. 2022). Parents would like contact with healthcare professionals to be characterized by openness, collaboration, and trust (Simes et al. 2022) and would like healthcare professionals to be non-judgmental, caring and taking the child and their safety seriously (Stewart et al. 2018). Apart from the child getting appropriate care, parents may also wish to receive professional help to manage their own feelings as well as support to improve the relationship with their child (Simes et al. 2022).

In psychiatric inpatient settings, gatekeeping structures and conventional practices surrounding psychiatric admissions may render iatrogenic harm such as aggravating self-harm (James et al. 2012; Burrin et al. 2021; Beale 2022; Griffiths, 2022). Brief Admission by self-referral (BA) is a standardized, brief, recovery-oriented form of admission intended to prevent and deescalate suicidal thoughts and self-harm impulses and prevent iatrogenic harm (Liljedahl et al. 2017a). The individual is empowered to self-admit at their own request for up to three days, maximum three times per month. They are encouraged to do so early on, and supported in understanding their own health and care needs so that crises may be prevented. BAs are made available through contract negotiation. Nurses and nurse’s aides manage the BAs and are trained to have a warm, open, validating and encouraging approach (Liljedahl et al. 2017b). In adult psychiatric settings, self-harm seems to decrease over time for individuals with access to BA (Westling et al. 2019) and BA is generally well-received by individual users (Lindkvist et al. 2021) as well as healthcare professionals (Lindkvist et al. 2019), associated with strengthened dignity and recovery for the individual (Helleman et al. 2018; Lindkvist et al. 2019, 2021). In child and adolescent psychiatry (CAP), legal guardians or other key adults partake in negotiating and signing the BA contract and are involved upon intake and discharge, but other than that, the intervention is aimed at the teenager. Differently from conventional admissions, parents or other key adults are not required to stay at the unit during BA; whether or not they stay is up to the teenager (Lindkvist et al. 2022). Having access to BA is in itself associated with decreased visits to psychiatric emergency care and decreased admissions for teenagers (Johansson et al. 2023). Teenagers describe feeling safe when having access to and using BAs, able to save themselves and ease the burden on parents and loved ones when they self-admit (Lindkvist et al. 2022). For more information about BA in CAP, see Lindkvist et al. 2022; Liljedahl et al. 2023.

Parents’ experiences of BA remain under-researched. One recent, phenomenologically oriented study (Hultsjö et al. 2023) describes the experiences of the surrounding family of adults with access to BA, where such access is described to bring about respite and hope for an improved everyday life for family members. No research exists, however, on parents’ experiences of BA within a CAP setting, yet such research is highly relevant given the uniquely preventive approach of BA and the strains and struggles faced by parents of teenagers who recurrently self-harm and experience suicidal thoughts. The purpose of the present study is to explore parents’ lived experiences of their teenagers’ use of BAs and elucidate the essential meaning of the BA phenomenon for the parent.

## Method

### Design

This is a qualitative study utilizing the phenomenological psychological analysis method (Englander & Morley 2023), which is based on Giorgi’s descriptive phenomenological approach to psychological research (Giorgi 1985, 2009). Phenomenological psychology was deemed especially appropriate to illuminate in-depth what it is like for the parent when their teenager self-admits with BA.

### Setting

BA is offered at a university hospital in Malmö at the only CAP inpatient clinic in Skåne, the southernmost region in Sweden with a total of 1.4 million inhabitants including 300.000 children. The clinic caters to children under 18 years of age and has two units. Teenagers self-admitting with BA share space with those admitted on voluntary and compulsory emergency admissions. There are eleven beds in total at the clinic, two of which are earmarked for BA. Admitted teenagers might reside anywhere in the region and may have their outpatient care connections at any of its 23 outpatient units.

BA is only offered to teenagers who have been in contact with psychiatric emergency services, meaning that most are struggling with both suicidal thoughts and self-harm and have previously been admitted with emergency admissions managed by a psychiatrist (such voluntary admissions are henceforth referred to as *conventional admissions*).

### Participants

Initially we wanted to offer the possibility of participation to all parents and other key adults of the teenagers who had access to BA. We invited 70 individuals, including biological parents, foster parents, parents’ partners, second-degree relatives and, when relevant, staff at special service housing. Out of these, 26 individuals agreed to participate, and we interviewed them all. However, we struggled with delineating a directly lived phenomenon in some of these 26 interviews. The child’s *access* to BA often appeared quite abstract in the interviewees’ awareness and was not necessarily something they concretely experienced at a given moment. As the most fundamental inclusion criterion in phenomenological psychology is that the participant has directly experienced a situated phenomenon (Englander & Morley 2023), we narrowed our inclusion criterion to parents having experienced concrete situations when the teenager had *used* BAs, a phenomenon that interviewees could talk about in detail.

Finally, then, our sample consisted of 17 parents who had experienced their children using BAs at least once. Thirteen were biological mothers, three were biological fathers, and one was a foster parent who had a longstanding relationship with the teenager. The age range of the participants was 38–60 with a median of 48 years.

Twelve parents had more children in the household, most often younger siblings. Nine of the parents lived with a spouse or partner, while six participants were the only parental figure in the household. In one case, the parent shared home and caretaker responsibilities with her own parent. In another case, two parental figures very recently separated into two households to be able to devote appropriate attention and care to each of their two children.

Five parents reported a personal history of mental illness, two of which mentioned history of self-harm and one mentioned personal experience with psychiatric admissions in her youth. Another reported having such experience through a sibling. Participants were not asked about this, but some offered up this information spontaneously. For a summary of participant characteristics, see Additional File 1.

### Procedure

Participants were recruited by phone calls to all key adults having signed a teenager’s BA agreement, active as of December 2021. When contact information was unretrievable using the contracts alone, the BA coordinator looked through patient records. When only one adult was listed, RL asked this person about others who perhaps ought to be offered participation. Everyone showing interest received written information about the study via email, then RL sent out a reminder email and attempted another phone-call once after about three and five weeks, respectively. Individuals unreachable at this point were considered to have declined.

During a period of the recruitment phase, due to the covid-19 pandemic, participation was only offered remotely via video or phone. When restrictions surrounding the pandemic were alleviated, participants were free to choose to be interviewed remotely or face to face at a private location of their convenience.

The interviews were conducted in December 2021-May 2022. For some interviewees, the teenager had only just received the BA contract a few months ago, whereas some teenagers had had access to BA for several years at the time of the interview (see Additional file 1). Participants were interviewed individually by RL. Before initiating interviews, RL repeated information about the study, answered any questions, and obtained participants’ written or recorded verbal consent.

Three interviews took place in in-person meetings in the participants’ homes. One interview started as a video meeting but, due to technical issues, was carried out over the phone instead, recorded over speakerphone. The others were video interviews, recorded in the Zoom video service provided by Lund University. One interview had to be interrupted after 22 min as the parent needed to respond to their teenager experiencing crisis. The other interviews lasted between 38 and 93 min, with a median duration of 55 min.

The interviews were semi-structured, supported by an interview guide. The interview guide was designed to facilitate narration of participants’ lived experiences, with particular attention paid to the existential constants of *body*, *time*, *space*, and *relations*, though interviews also covered *world/reality* and *sense of self* (Englander & Morley 2023). Questions included, for example, ‘How do you spend your days when [your child] is admitted with Brief Admissions?’, ‘Have you ever been present [at the psychiatric unit] during a BA? What has that been like?’ and probes for examples of concrete everyday events. The guide was developed by the research team and reviewed by a representative of the Swedish Partnership for Mental Health (NSPH), a non-governmental organization working for increased participation of patients and family in mental healthcare. Following review and revisions, the interview guide was tested in a pilot interview. No further adjustments were necessary, and the pilot interview was included in the analysis for this study.

### Data analysis

RL was the driver of the analysis process, which four of the authors were involved in.

First, the authors read through each transcript to get an initial sense of the whole. We adopted a phenomenological *psychological* attitude, practiced epoché (that is, we set aside our preconceptions of the phenomenon as objectively, independently existing in the world), and practiced (psychological) reduction to narrow in on the psychological region of meaning in the parent’s lived experience. We then identified all sections related to the teenager’s use of BAs and divided these into their smallest meaning units in a new document. In a column beside these original verbatim expressions, we elucidated the psychologically relevant meanings of each of them, seeking to avoid unwarranted interpretive leaps or premature exclusions. RL then wrote cohesive, rich yet prudently concise texts explicating the (idiographic) psychological meaning structure of each parent’s lived experience, with no between-subject comparison. Refocusing on the existential constants and practicing free imaginative variation opened up to new intuitions of the whole. Finally, we moved from the vastly different situated structures to a general meaning structure of the phenomenon, again using imaginative variation and removing everything that did not appear essential. No direct participant quotes were included in this final text, as participants do not speak directly to the general level of meaning explicated by the researchers (Englander & Morley 2023).

In sum, we moved from whole to part to whole to arrive at an understanding of how the meaning of lived experience was structured. Throughout the process, the authors involved in the analysis met regularly for in-depth discussions and repeated revisions. All five authors were involved in revising the results.

## Results

Our participants experience their teenager’s use of BAs in markedly different ways. We elucidate here two distinct meaning structures of this experience: ‘being gifted relief and hope’ and ‘being robbed of everything you believed in’. For each essential structure, we will first present its full gestalt, then explicate its constituents and their interrelatedness in depth.

### Essential meaning structure: being gifted relief and hope

Their teenager using BAs is a blessing in the parent’s experience. In a dangerous world, the parent rests assured that their teenager is safe during BAs. They feel a wave of relief, no longer needing to live their life in a constant state of emergency. The parent is liberated; their autonomy increases along with the teenager’s during BAs. Everyday life begins to feel recognizable and normal for the whole family, bringing back hope for a future. The teenager grows increasingly independent, and the parent trusts them to manage their own health(care). As the teenager steps up, the parent steps down. This transition sometimes feels painful and difficult, yet the parent accepts it as healthy. Being supportive from a distance, the parent increasingly gets to be their own person in relation to their teenager.

#### Constituents of ‘being gifted relief and hope’

In the essential structure of experiencing BAs as being gifted relief and hope, the following constituents reveal themselves: ‘a sense of safety and containment’, ‘liberation from a hostage situation’, ‘a return to wellbeing’, and ‘catalysts for relational shifts’.

##### A sense of safety and containment

Having a teenager who self-harms and experiences suicidal thoughts, the parent has been living in fear for their life. The lived world is one full of dangers: the city full of parks where the teenager may run off, of stores selling potentially harmful items, and of people who may have sinister intentions. To some extent, the parent still relates to the world this way during their teenager’s BAs, especially since the teenager is free to come and go. Even so, the parent relates to the BAs as safe for their teenager – *uniquely* safe, in contrast to the controlled environment during conventional admissions, which is accompanied by a sense of unpredictability, incomprehensibility, and imprisonment.

The BAs also feel safer than the family home. The house is a potential death-trap where locked medicine cabinets may be pried open, cleaning aides may be consumed, and a whole host of everyday objects may have unpredictably sharp edges. The parent feels out of control of their own space, unable to mitigate perils, sometimes helplessly watching as their teenager self-harms. Difficult experiences from the past may also haunt the house, etched into the walls and floors like hidden mines, so that the lived space manifests as even more hazardous, where the parent gets no rest.

From this place, the parent is relieved when the teenager self-admits. The parent knows their teenager will be kept safe during BAs, in a low-stimuli environment where professionals look out for them and tend to their basic needs. Whether there with the teenager or not, the parent feels their teenager is *contained* in a positive sense: being held, getting to take a break and simply be. The parent feels contained as well, given a cue that they may now, finally, let go of their worries.

##### Liberation from a hostage situation

Prior to the teenager using BAs, the parent has their temporality constrained by the incessant need to be ready to respond to crisis. This makes for an everyday life jagged by interruptions; the parent suddenly needs to leave work, drop the employment interview, cancel family activities and any personal plans – essentially drop everything to speed to their teenager’s rescue. Life is moving from exception to exception with no sense of continuity or constancy. The rest of the world ceases to exist.

The parent is spatially arrested as well. They stay at home to watch over the teenager, surveilling their every move as their warden. The price to pay for this is high; the parent cannot sleep properly, tend to their bodily needs, go outside or be with others. The house is stripped of its foundational homeliness and takes on an atmosphere of entrapment for both teenager and parent. This feeling lingers during the teenager’s conventional admissions as the parent is detained in the psychiatric unit, forced to breathe down their teenager’s neck against both of their wishes. Even if permitted to go home briefly, the parent remains in lockdown, tensely waiting to be summoned back. Sometimes, this hostage situation extends to their other children, unable to leave the house not knowing when their parent will be back.

The parent may experience their sense of self held hostage, in the strain between parental responsibilities for their sick teenager and other responsibilities, such as work or tending to the needs of siblings. Younger siblings may witness frightening episodes during crisis, older siblings may tackle the teenager in crisis like security guards to protect them, and amidst this turmoil the parent’s heart breaks. Being a good parent, professional, person appears impossible. The parent experiences internal and external blame, guilt, and shame for not doing or being enough.

When the teenager starts using BAs, however, everything changes. The parent regains spatiotemporal agency; they can take off their soldier’s helmet and step down from their exhausting, all-demanding stand-by duty. This liberation marks a return of autonomy and agency for the parent as well as the teenager. The parent is also free to have a dialogue with the teenager about their preferred degree of contact at the psychiatric unit, free to give the teenager room (to decide). The parent’s sense of self (as parent) is relieved from threat and, in accord, tensions within the family dissipate.

##### A return to wellbeing

The BAs represent a turning point for the parent: before them, life is suspended in space and time; with them, life begins anew. A burden is lifted and the lived body physically de-stresses, shoulders relaxing, lungs inhaling deeply and releasing a sigh. The parent associates their teenager’s BAs with bodily self-care practices, like having a massage.

Little by little, the parent sees the teenager learning to care for their own emotions and needs, in a way they haven’t before. Even if they might still be struggling, the world and life itself begins to feel manageable for teenager and parent. Throughout crises at home, contact with psychiatry and conventional admissions, the whole family has collectively been sharing feelings of anxiety, frustration, despair, and hopelessness. When the teenager uses BAs, instead, the parent feels that the family shares experiences of relief, safety, calm and hopefulness. The siblings go on being children and the parent is more continually and predictably available to them now, getting to spend some treasured quality time with them. The BAs assimilate into and support a familiar family life, bringing back a sense of normalcy that the parent celebrates.

The parent can also return to familiar activities and settings. Going back to work is a huge positive leap, not only in terms of escaping the stress of leave-of-absence and stabilizing the family’s economy, but in terms of gains intimately connected with a sense of a healthy life, like feeling gratitude and meaningfulness when tapping into the roles of the professional and colleague. These identities bring a sense of wholeness to their selfhood and allow them to reconnect with social relations, receive invaluable support from colleagues, take part in other people’s lives and feel like they belong.

##### Catalysts for relational shifts

Both teenager and parent need to adjust to a new way of thinking. With psychiatry suddenly encouraging the teenager to initiate admissions, and enabling them to choose to admit alone, the parent’s lived experience with the world shifts to one where help is available, and they do not need to be the one to manage everything. These circumstances catalyze a shift in the parent’s lived relation with their teenager. As the teenager starts experimenting with how the BAs might be helpful, the parent gives them space and encouragement.

Stepping back from managing, moderating, and monitoring is challenging, though. Adjusting to a new world order where the teenager is not in imminent danger, the parent finds comfort in the realization that taking a step back is challenging to *all* parents – and a normal and healthy part of parenting a teenager. Equally normal is the sometimes-painful experience of not being needed as much, not getting to perform the same concrete acts of care anymore. The parent retains their sense of self (as parent) though, adjusting to a new parental role.

The sense of normalcy affords the parent with faith. They choose to trust their teenager, the professionals, and the BA method. They no longer need full insight into the happenings at the psychiatric unit and feel comfortable not being there. Their spatial absence manifests as freedom rather than hindrance, a natural part of a healthy process, and the parent willfully surrenders control and responsibility. The more the parent can let go, the more they get to experience their teenager growing and maturing, gaining self-understanding and self-regulation skills.

The parent attunes to where the teenager needs them to be. They acknowledge the teenager’s capabilities, validate their need for space, mirror their disappointment in the face of setbacks, and encourage them. In place of the previously hierarchical parent-child relationship, a more symmetrical relationship is forming. The parent starts distinguishing the teenager’s needs from wants and preferences and can begin to consider their own as well, taking shape as person-beyond-parent even in relation to the teenager. The BAs provide parent and teenager with time and space to relate differently to each other.

### Essential meaning structure: being robbed of everything you believed in

For the parent, in using BAs their teenager is being deprived of their fundamental needs. The parent engages in a tug of war for control where there is no true winning. The physical environment feels like a wasteland, a space submerged in despair and indignity, robbing the parent of hope. The psychiatric staff feel unreliable, inexplicably abandoning the teenager during BA. The parent does not see the purpose of the BAs, they seem like an echoing void where the teenager receives no help. Nothing adds up, nothing makes sense; there seems to be no thought behind anything. The parent is caught in the present, though aching for a future. They expected more from the BAs and psychiatry at large.

#### Constituents of ‘being robbed of everything you believed in’

In the essential structure of being robbed of everything you believed in, the following constituents reveal themselves: ‘a tug of war for control’, ‘an unworthy wasteland’, ‘abandonment and collapse of authority’, and ‘no sense of purpose and plan’.

##### A tug of war for control

The teenager’s life has been hanging in the balance for a long time. Under such grave circumstances, the parent expects the psychiatry professionals to remain in control over the teenager at the unit. But the teenager is free to come and go during BA and the staff seem to have no clue about their whereabouts. The parent is given no reason to have faith in the BAs, and in their mind’s eye they are bombarded with dire scenarios of their teenager self-harming or running away. The parent’s lived sense of self is that of a rock, the one dependable thing out there, and they simply cannot take the risk of surrendering control to no-one.

The parent engages in a tug of war versus both the teenager and the healthcare system. While the teenager is happy about their strengthened voice and autonomy, the parent does not trust their teenager to understand their own health and care needs or make wise decisions during BAs. Sometimes the parent is the one to get the teenager (self-)admitted. Sometimes the parent speaks for the teenager during intake meetings, or over the phone in the teenager’s absence. It may be that the teenager actively seeks the parent’s support, but the parent may also feel the need to go over their head in making plans about what is best for them. The parent instinctively cares for their teenager as if they were a younger child.

The tug of war with psychiatry means fighting both people and system. Paradoxically, the parent may authoritatively scold or command the psychiatry professionals, not trusting their judgement, yet insisting that the teenager is safer at the psychiatric unit than at home. But the parent must submit to certain conditions set by the psychiatric system, such as not staying at the unit and not listening in on conversations with professionals if the teenager doesn’t want them to. The parent feels distressed about this loss of insight into the teenager’s admission, not getting to experience first-hand that their teenager is safe. The parent’s lived space of detachment and absence rules them while they make every effort to overrule it. The parent is distinctly *not-there*, so painfully on the *outside* physically that they must always remain *inside* mentally. They frequently call the unit and text their teenager, they worry incessantly, and may even try reaching the teenager’s friends for updates. ‘Winning’ the tug of war changes little of the teenager’s situation, but losing is excruciating.

##### An unworthy wasteland

When accompanying the teenager to the psychiatric unit, the parent faces an environment conveying scarcity and urgency. Children’s suffering infuses the walls and hovers in the air, subtly yet profoundly weighing down on the parent, making them feel heavy, uneasy, suffocated. The other children admitted are also perceived as threats who may disturb their teenager’s delicate balancing act between health and illness, triggering self-harm or an eating disorder and competing for the attention of busy professionals in a setting plagued by high turnover and chaos. The parent instinctively wants to escape this environment yet may also feel a need to control it.

The room that is the teenager’s intended refuge is like a prison cell or broom closet – a tight, barren, inhospitable and unpleasant space devoid of hope. The teenager may be left with a bare mattress on the floor. The sense of unworthiness oozes from the lived space that is this wasteland. Though the parent may try to improvise curtains from blankets, the spatial inhospitality is difficult to challenge. The devoid setting, in its silence and givenness, has a demonstrative property to it, declaring *this is the world you live in now; this is all there is for your child*. The parent’s heart sinks.

##### Abandonment and collapse of authority

Sometimes as the teenager self-admits alone, soon enough the parent starts receiving messages that their teenager feels alone. Maybe they have left the unit, sitting isolated in some diner. It tugs at the parent’s heartstrings to imagine their teenager lonely and abandoned like a street child, especially while feeling poorly. The parent’s instinctive response is to bring them home immediately, and so the *why* of the teenager’s being there-not-here is actualized. The implicit agreement to temporarily outsource the care of the teenager from parent to professional, is now violated. The parent experiences a relational breach of trust, betrayal, and is filled up with rage. How can the staff simply leave the teenager? How can they not *care*?

The inaction and passivity of the professionals is interpreted either as incompetence and clumsiness, or as neglect and unforgivable recklessness. It feels deeply unethical, borderline criminal, to leave a suicidal teenager to their own devices. These experiences hollow out the authority of the professionals.

In fact, the nurses and nurse’s aides are not regarded as professionals at all. The parent is more inclined to trust psychiatrists, psychologists, and healthcare counsellors, feeling that they represent *real* care. The parent is more oriented toward their absence than the presence of other professionals during BA. The contributions of the professionals present are eyed with skepticism, if at all considered. When the parent stays at the unit with the teenager, they sense that the staff consider BA patients less worthy of care, like second-class patients, deprived of their rights. Not feeling taken seriously makes the parent not take the BAs seriously. The professionals’ betrayal is experienced as deeply personal and unrecoverable.

##### No sense of purpose or plan

The parent feels frustrated as the BAs don’t seem to make any tangible difference in the family’s life. They seem pointless, futile, and impractical, or their short-term helpfulness appears to hinge on coincidence, such as which members of staff happen to be on duty at the time. Their teenager is in a no-man’s-land, a trench where no one will claim them, contained like an object.

The parent grasps for something to believe in, feeling a pressing need to comprehend the overarching purpose with the BAs, get a sense of direction, a plan for their teenager. But the professionals handling the BAs offer no insight into the bigger picture. The parent’s lived time is in a standstill. They are arrested in the present situation, their future a thick fog, ethereal and impossible to see through.

The parent is left feeling that they expected more from the BAs and psychiatry. They have been to war for ages to get their child the healthcare they need. This care should feel like an all-hands-on-deck situation, but the psychiatric system is deceitful and undependable. In the parent’s lived world, the BAs are bookkept along with other disappointments, as a budget alternative to *real* care for a healthcare system on its knees. Realizing that the glimmer of the BAs was really *l’or des fous*, the parent feels disillusioned. Certain that their teenager would not survive losing hope, the parent keeps their soldier’s helmet on – they cannot and will not lose this fight.

## Discussion

The present study elucidates what the teenager’s Brief Admissions may be like for the parent, experienced as being gifted relief and hope or being robbed of everything you believed in. Given the purpose and delivery of the BA intervention as studied here, the parent’s experience of the BAs will inevitably be nested within their experience of having a teenager who self-harms and experiences suicidal thoughts. Indeed, we recognize several elements of our present findings from previous research on being a parent under such circumstances.

The parent being on the alert for dangers in the home environment and attempting to control them, as seen in the constituent *A sense of safety and containment*, is a well-documented experience for parents of children who experience suicidal thoughts and self-harm (Daly 2005; McDonald et al. 2007; Steggals et al. 2020). This may be thought of, in Heideggerian terms, as a shift of the parent’s Being-in-the-world: they return to a state of hyper-attunement with their child, a parental worldhood where mortal danger appears to be lurking at every turn in the everyday environment, a way of being which is usually reserved for the child’s infancy and toddler years (Darbyshire & Oerther 2021). Notably, when the BAs are experienced as being gifted relief and hope, one key to this experience is that the BAs offer a safe environment for the teenager and relief for the parent, a break from needing to worry about perils at home.

Further, our finding that the parent of a suicidal teenager lives in constant fear and preparedness to respond to crisis, is in line with previous research (Daly 2005; Buus et al., 2014; Weissinger et al. 2023; Hultsjö et al. 2023). The present study also reaffirms findings of the parent’s guilt about not being enough for the teenager who self-harms and/or experiences suicidal thoughts (McDonald et al. 2007; Buus et al., 2014; Weissinger et al. 2023; Krysinska et al. 2020; Hultsjö et al. 2023), as well as the guilt and strain of not being able to properly live up to other responsibilities, such as work or tending to one’s other children (Daly 2005; McDonald et al. 2007; Buus et al., 2014; Curtis et al. 2018). This feeling of pressure and failure is also related to a loss of sense of self and a de-prioritization of the parent’s own needs, as seen in the present study and others (Daly 2005; Krysinska et al. 2020; Juel et al. 2023; Hultsjö et al. 2023). Related to this last point, Husserl uses parenthood (motherhood) to exemplify the absolute value of interpersonal relations, one that trumps other values, commitments, and desires the individual might have (Leon-Carlyle 2021). Crucially, the present findings reveal how the parent may be liberated from guilt, strain, and loss, and regain a sense of predictability, agency, and selfhood beyond parenthood in everyday life while the teenager uses BAs. The teenager’s BAs may construct for the parent a new situation in which their self-constitution as parent, their dedication to the absolute value of caring for their teenager, can be upheld without requiring them to sacrifice themselves.

Also noteworthy, particularly in the constituent *A return to wellbeing* is how prominent aspects of the experience of the teenager’s self-harm, suicidal thoughts, and recovery are shared within the whole family system. Parent and siblings experience anxiety and despair, frustration and hopelessness together with the teenager through crises and psychiatric contacts. Not only does the parent witness the teenager’s relief, hopefulness, and recovery during BAs; they experience this first-hand as well, as they recover a sense of a normal life. From this, recovery is not only an individual process but a social one involving the entire family, as noted recently by Weissinger et al. (2023). Here we might draw parallels to the Husserlian sense of human consciousness as fundamentally interpersonal, for no one could ever experience a world as anything but ‘a world pregiven to [them] in [their] conscious life and in community with fellow human beings’ (Husserl 1970, p. 165). This position is at the heart of Davidson’s (2018) account of the social embeddedness of recovery. The social context of the family shapes the teenager’s (ill)health and may support growth and recovery, and beyond that, recovery can also be understood as social in the sense of experiential sharing (León & Zahavi 2016), a collective experience of which the parent is not merely part, but subject. Attentive to this dual role of the parent in the process of recovery, we add another voice of critique against reductionist, individualist explanatory models of mental illness, aligning this study with the longstanding critical tradition of phenomenological psychiatry (Zahavi & Loidolt 2022).

Relating to the constituent *Catalysts for relational shifts*, this is at once also a critique against the notion of *re*covery as returning to a baseline state ‘before’ ‘illness’ (Davidson 2003), as beyond recovery, we see that the BAs may be sites of *un*covery: the teenager uncovers independence and personal growth not previously experienced, while teenager and parent uncover novel, healthy ways of relating to each other. Their ability to communicate around self-harm, with the parent asking about the teenager’s needs and preferences in relation to the BAs, is contrary to the interpersonal communicative breakdowns observed in Steggals et al. (2020) and Curtis et al. (2018). Certainly, interpersonal communication might be stimulated by a joint focus on the healthcare setting and care decisions at large, as indicated by the parental communicative tendencies reported in Lindgren et al. (2010).

*Being robbed of everything you believed in* speaks of entirely different ways of meaning-making in experiencing the BA phenomenon. The parent does not trust the BA method and is fighting psychiatry. We concord with Weissinger et al. (2023) that parental loss of control is terrifying, perhaps even traumatic, when one’s child wishes to die and one cannot make sense of the healthcare system. Indeed, a developmental regression in parenting characterized by retracting the teenager’s independence and increasing control over them, is a known tendency among parents of children who self-harm (McDonald et al. 2007; Curtis et al. 2018; Hultsjö et al. 2023), and finding a good balance between enforcing boundaries and being permissive and open appears to be challenging (Krysinska et al. 2020). In our study, parents experiencing *A tug of war for control* are struggling with this, while parents experiencing *Catalysts for relational shifts* appear to have been able to uncover a new balance in their parenting, guided by trust. This is an exciting finding, although we must acknowledge that this individual-relational maturation process might have occurred for these parents and teenagers even if the teenager would not have self-admitted with BAs. Even so, the BAs appear to have potential in supporting healthy development.

Relating to the physical space at the psychiatric inpatient unit as a prison, and perceiving the other children admitted as potential threats to the parent’s teenager, are experiences observed in this study and previous research (Lindgren et al. 2010; Weissinger et al. 2023). This ties in with experiencing contact with healthcare professionals as a struggle, feeling that one’s child does not get enough or appropriate care, not feeling considered as a parent or feeling actively excluded from partaking in the care of the child (Lindgren et al. 2010; Simes et al. 2022; Hultsjö et al. 2023), experiences that we also found in the present study. Such experiences, on top of the original distress of the child’s self-harm and/or suicidal thoughts, may jointly influence the parent in not recognizing the nurses and nurse’s aides as professionals. These experiences are all clearly stressful for the parent. Particularly in *A tug of war*, we see how the struggle with exclusion and being on the *outside* of care becomes existentially acute for the parent. Previous phenomenological works may help us understand the magnitude of existential crisis, as parents of hospitalized children have a foundational need to *presence themselves* with their child, to be-with them and keep vigil by their side (Darbyshire 1994), to stand by, bear witness and sink down into the child’s sphere of existence (Darbyshire & Oerther 2021).

A final noteworthy finding is that the parent may view the psychiatric system itself as ‘sick’. They may interpret the relative absence of interventions and professional interactions during BAs as further evidence of their prior experiences of psychiatric care as *devoid of care*, stripped of financial resources and plagued by a short-sighted mechanistic view of patients as objects to handle and store, rather than human identities to relate to. There is a deep, seething sense of injustice here, which comes across as built into the very healthcare system. In contrast, in the other variant of experiencing the BAs as being gifted relief and hope, hopefulness may be associated with the sense that individuals who self-harm and experience suicidal thoughts are seen as persons with voice, abilities, agency and lives to lead. This is testament to how BA might be one potential ingredient for an antidote addressing structures of injustice within psychiatry (Scrutton 2017; Ritunnano 2022), entrenched within deeper political and economic conditions of oppression and privilege in society at large, that reify illness and maintain objectification, passivity, and subordination of those turning to psychiatry for care and support (Basaglia et al. 1987). Certainly, such societal issues extend beyond the psychiatric system. Notably, parents experience barriers to care, such as perceiving that healthcare providers do not offer adequate support, communicate, listen to, consider, or involve parents within the care setting, also in community-based alternatives to inpatient psychiatry (Vusio et al. 2020). A promising development within psychiatry is the turn toward shared decision-making and emergent efforts to incorporate parental involvement into such a framework, which may help parents support their children toward gradually greater independence (Bjønness et al. 2022).

### Clinical implications

The above discussion points to the health-promoting potential of BAs for the parent and the whole family, as well as for the teenager who is the target of the intervention. We note the important role of mental health professionals in realizing this potential, in terms of their interaction with teenager and parent during BAs and importantly also in framing these admissions to the parent. It is imperative that professionals working with BA in CAP settings are attuned to the parent’s experience of the world in relation with their teenager. Such an attunement would enable the professional to, carefully and tactfully, support the parent’s sense of coherence; the parent needs to experience their teenager’s BAs as comprehensible, manageable, and meaningful (Antonovsky 1987).

In line with previous research (Simes et al. 2022), we reaffirm the parent’s need for professionals to be open, transparent, and collaborative. As has been made abundantly clear in the present study and previously, the parent needs to trust healthcare professionals (Simes et al. 2022) and needs them to take the child and their safety seriously (Stewart et al. 2018). Notably, it appears to be in the context of incomprehensibility and lack of meaning that the parent may feel as though the BAs are not ‘real’ care and that the nurses and nurse’s aides managing the BAs are not ‘real’ mental health professionals. Professionals working with BA may need to work even more actively on establishing trust and a sense of coherence. This could be done, for instance, by devoting some time to talk proactively to the parent about potential struggles that they, as parent, may encounter during future BAs. Of particular importance, they should address how it may feel stressful and counterintuitive for the parent not to be physically present during BAs, in contrast to conventional admissions. While the explicit request to leave is likely to be posed by the teenager, it might as well have come from the professional as representative of the BA method. The professional enables and accepts this request, ultimately harboring the *expectation* of the parent to comply. Conveying this expectation without acknowledging the parent’s context – as if it is a given that the parent is suddenly ‘not to worry themselves’ any longer – would be insulting and insensitive, completely overlooking the existential cleft that is ripped open for the parent.

To help bridge that cleft, professionals may do well to emphasize that they are indeed oriented to the teenager’s safety and are in no way down-prioritizing them. We also recommend that professionals reiterate for the parent the rationale behind certain BA procedures, such as why the teenager’s medical prescriptions will not be adjusted and why numerous professional appointments will not be imposed on them, reorienting the parent to a preventive, recovery-oriented mentality. The professionals’ views on how BAs might fit into the teenager’s life ought to be explicated, as well as the new role of the parent and how they may best support their teenager’s development (Zhao et al. 2022; Simes et al. 2022).

Parents may benefit from having such a conversation with mental health professionals in private and prior to the teenager’s first use of BAs, perhaps in connection with the BA contract negotiation. Professionals might also address these matters spontaneously as they come into contact with parents over the phone or in person during BAs; this would be especially important if professionals sense that a parent might be struggling with the BAs.

### Strengths and limitations

There are several frameworks for evaluating the rigor and contribution of qualitative research, where Polkinghorne’s criteria of accuracy, richness, vividness, and elegance are suggested for evaluating phenomenological research (Polkinghorne, 1983, cited in Finlay 2006). Accuracy in a phenomenological sense would mean that the phenomenon has been explicated in a way that the reader can recognize from their own lived experience or imagination. Vividness would be fulfilled if the reader is pulled into the reality of the phenomenon as lived, and relatedly, richness would indicate rich enough descriptions so as to engage the reader emotionally. Finally, elegance is about capturing the phenomenon in writing in a clear and graceful manner. We have certainly been guided by these criteria throughout the research process and, specifically in the analysis and summary phases, have sought to fulfill them; however, the reader is the ultimate judge of our degree of attainment.

Additionally, we have sought to adhere to recommendations for methodological integrity, in terms of fidelity and utility, specifically for qualitative research within the psychological discipline (Levitt et al. 2017), which has been conceptualized within phenomenological psychology as remaining faithful to the phenomenon and research goals by applying the constituents of research consistently and taking care not to let our presuppositions impede our phenomenological intuitions (Churchill 2022). To this end, we have adhered closely to our aims and methodology, including narrowing the focus of our study to *use* of BAs when we recognized that our original inclusion criterion was not well-defined for phenomenological research. We have practiced epoché and phenomenological psychological reduction continually. Overall, we have taken care to follow Englander and Morley’s (2023) recommendations and sought to be transparent and clear about our procedures, choices, and research process.

Further, traditionally in qualitative research we ought to provide rich information about the research setting and context of our participants in order to clarify the potentials and limits of transferability of our findings (Levitt et al. 2018). We have provided such information in this article, yet it should be mentioned that our phenomenological methodology, with structural analysis based upon the epoché and free imaginative variation, uniquely enables us to make claims beyond the situated experiences of our particular sample (Husserl 1970; Churchill 2022), an important strength of our study. Indeed, our descriptions of what the BA phenomenon may be like in the lifeworld of the parent are not direct summative reflections of the participants’ reported experiences, as phenomenological psychology has the (psychological) phenomenon as its object of study (Englander & Morley 2023; Churchill 2022). Few of the participants were immersed purely in one of these two ways of experiencing, and some did not have as strong experiences as are indicated in the phenomenological descriptions. In this context we also want to be clear that our studied phenomenon is related to delivery of a healthcare intervention at one clinic. We know that self-admission interventions are offered under the same or different names elsewhere (Eckerström et al. 2020; Moberg & Schön 2022; Hultsjö et al. 2023), using varying target groups and procedures, which may make for different lived experiences of different phenomena.

Again, the extent to which we have succeeded in generating essential descriptions of the general meaning structures of the BA phenomenon is up for reader verification. One prominent limitation of our study, arguably the greatest threat to our claims, is our limited ability to fully ‘see’ the phenomenon we seek to study (Churchill 2022). We consider our research team to be well-balanced, including individuals with extensive experience doing qualitative as well as psychological research, and individuals with varying relations to BA: as implementers, educators, clinicians, researchers, or having no previous relation to BA whatsoever. Additionally, some members of the team have their own lived experiences of being parents, others of being carers of loved ones who live with self-harm and suicidal thoughts. These experiences have all informed the study and to some extent aided our intuitions of the phenomenon. Yet it must be said that all members of the research team had limited previous experience with conducting phenomenological psychological research, which no doubt has clouded our vision. We have consequently attempted to familiarize ourselves extensively with foundational phenomenological literature, methods literature, and primary phenomenological psychological research.

Other issues that might have affected our ability to see the phenomenon pertain to the interview situation. Interviewing everyone who wished to participate may be regarded as a strength, yet it also meant we conducted vastly more interviews than would have been strictly methodologically necessary (Englander & Morley 2023), which made the analysis process incredibly time-consuming and resource demanding. In this situation, it was not feasible for us to conduct more than one interview per participant, although repeated interviews might have offered a deeper understanding of the phenomenon. Further, the semi-structured interview format was selected for its strength in offering both focus and flexibility, though in this case unstructured interviews might have elucidated more free narratives. Material prompts, like the BA contracts or a pre-composed written participant account of one time that the teenager used BAs, might have offered a focal point encouraging free narrative elaborations on experiences as lived. This might have also helped parents to focus back on their own experiences rather than (their beliefs about and attunement to) the teenager’s experiences, a distinction which participants commonly struggled with.

Even so, this study is uniquely informative of the parental lived experience of their teenager’s psychiatric admissions with BA, nested within the parental experience of their teenager’s self-harm and suicidal thoughts. Having performed a deep structural analysis of essential meaning, our research is grounded in human experience, which enhances the relevance of the recommendations we pose for improved clinical practice within CAP inpatient care.

## Conclusions

Being the parent of a teenager who recurrently self-harms and experiences suicidal thoughts may mean having one’s lifeworld reconstituted as a world full of perils, one where the parent must sacrifice life as they knew it in order to protect and fight for their teenager. The teenager’s BAs may become a turning point for the parent: a gift which helps the parent recover safety, familiarity, and agency in their everyday life, while uncovering new ways of being with their teenager. In this way, well-being may be enhanced for the whole family. However, the parent may also sense the promise of a turning point that does not arrive, leaving them feeling robbed of everything they believed in. A phenomenological psychological lens reveals the need to support parents who are struggling existentially, feeling unmanageably challenged in their parenthood during the teenager’s BAs. To do this, mental health professionals working with BA must mindfully, sensitively, and tactfully help reorient the parent to a sense of manageability, comprehensibility, and meaningfulness, preparing them for a new parental role. While BA educators may make effort to spread awareness among all professionals working with BA, representing continuity for families within a mental healthcare setting is a complex task, which may arguably be facilitated if professionals experience such continuity in their workplace. This may be achieved on different levels, from implementing routines and documentation helping professionals inquire about parents’ experiences in connection to BA contract negotiations, evaluations, and intake and discharge meetings, to dedicating supportive resources such as informative handouts or group psychoeducation sessions for parents whose children are provided access to BA, to more overarching workplace efforts to address work environment and employee turnover. Future research might explore the perspectives of healthcare professionals working with BA within CAP and how they relate to initiatives such as shared decision-making and person-centered care in the context of working with children and families affected by self-harm and suicidal thoughts and behaviors.

### Electronic supplementary material

Below is the link to the electronic supplementary material.

Supplementary Material 1

Supplementary Material 2

## Data Availability

The data that supports the findings of this study contains sensitive information and is not publicly available due to the risk of compromising participants’ privacy. Even in the case of de-identified interview transcripts, the aggregate of multiple pieces of such information may still risk compromising participants’ confidentiality. Participants did not provide informed consent for data sharing and the current ethical approval by the Swedish Ethical Review Authority (Reg. No. 2020 − 01840) does not cover data sharing. As such, neither the transcripts nor any form of partially curated data will be made available.
