# Supplementary material for: Receiving a gift and feeling robbed: a phenomenological study on parents’ experiences of Brief Admissions for teenagers who self-harm at risk for suicide
Source: Child Adolesc Psychiatry Ment Health. 2023 Nov 8;17:127. doi: 10.1186/s13034-023-00675-y (PMC10633972; doi:10.1186/s13034-023-00675-y)
Supplement: Supplementary file 1 — Supplementary Material 1 [file 13034_2023_675_MOESM1_ESM.docx]

*Table 1. Participant characteristics and contextual information**

| **Participants’ ages**  35-40  41-45  46-50  51-55  56-60 | 3  4  5  3  2 |
| --- | --- |
| **Participants’ genders**  Women  Men | 13  4 |
| **Parenthood**  Biological parent  Foster parent | 16  1 |
| **Ages of children with BA contracts**  14-15  16  17-18 | 4  7  5 |
| **Legal genders** of children with BA contracts**  Girls  Boys | 13  3 |
| **Time since child received access to BA**  Up to 6 months  > 6 months – 1 year  > 1 year – 1.5 years  > 1.5 years – 2 years  > 2 years | 4  4  5  1  2 |
| **Number of siblings (number of households)**  Younger siblings in household  Younger siblings out of household  Older siblings in household  Older siblings out of household | 12 (10)  4 (2)  3 (2)  14 (8) |

* All data on participants as well as children are based on parental reports, unless otherwise specified.

** Legal genders of children were inferred through their Swedish personal identity numbers, representing gender assigned at birth; however, we do not have information on children’s self-reported gender identities.
