# Supplementary material for: Receiving a gift and feeling robbed: a phenomenological study on parents’ experiences of Brief Admissions for teenagers who self-harm at risk for suicide
Source: Child Adolesc Psychiatry Ment Health. 2023 Nov 8;17:127. doi: 10.1186/s13034-023-00675-y (PMC10633972; doi:10.1186/s13034-023-00675-y)
Supplement: Supplementary file 2 — Supplementary Material 2 [file 13034_2023_675_MOESM2_ESM.docx]

*Table 2. Excerpts from the analytical process**

| *Verbatim expressions* | *Initial psychological elucidations* | *Further elucidations* |
| --- | --- | --- |
| **Interviewer**: Um, what has it been like for you during that time when [your child] has been admitted [with Brief Admissions]?  **Participant 3**: No well then I know- like, then I can relax. That’s what’s so good, then I can relax. When, and- Uh… The difference is also that when she’s been in on an emergency admission, then there are lots of visits with doctors, visits with psychologists, visits with healthcare counselors, so that… Everything is being put on hold, when [my child] was admitted on regular emergency admissions. I couldn’t do anything, the children couldn’t do anything, those who were at home they were still on-call as well. […] Because they know that I have to go, because all of a sudden they will call me from the psych emergency unit and go ‘hi, it’s 9:30 now, we would like you to come in at 10:15 for a meeting. And then we have another meeting at uh, 15:15 also.’ […] So it was always that I had to… I hardly dared to go to the bathroom in the end because they can call and tell me ‘now you are going to a meeting’. And I had to call my job and say ‘no I… [pause for 2 seconds] I can’t work. My daughter is admitted and I have all these meetings and…’ [sigh] But when she has Brief Admission, there are no meetings! No meetings! [My child] handles that all on her own. […] She has- There are like no, not a bunch of assessments, no doctor’s appointments, she can go talk to someone if she wants to but she takes care of that all on her own. | Participant 3 describes that she can relax when the child is admitted with BA. She contrasts this to experiences of conventional admissions, which she relate to as disruptive for the family’s everyday life, due to frequent meetings and the requirement of parental presence. The mother describes that she and her other children ’were on-call’ during such admissions, that nobody could do anything while tensely waiting for the mother to have to go to the psychiatric emergency unit. Participant 3 described it as hardly daring to go to the bathroom while her child was conventionally admitted and that she could not work during such periods. During BAs, instead, everyday life is sustained for the rest of the family. The child may receive supportive conversations during BA, not requiring parental presence, and so the mother and siblings have regained their freedom to decide over their own time. | The parent’s lived body is tense and weighed down during the child’s conventional admissions, preventing the parent from taking care of their own bodily needs. But during BAs, the lived body finds relief and relaxes.  Time and space, for the parent and other family members, are not lived as belonging to them during conventional admissions. The family is not free, rather held hostage in their own home.  During BAs, in contrast, the family is free and experience continuity in their everyday life. |
| **Participant 7**: I give her a ride [to BA]. But I’m absolutely not allowed to accompany, accompany her inside. Uh, but rather you’re supposed to leave her outside, uh…  **Interviewer**: Mm. [pause for 2 seconds] Right. So you drop her off, and…?  **Participant 7**: [pause for 2 seconds] Yes [sigh] Some, the time before last time I think it was, then I went- I wanted to accompany her to the door and then she got really angry. But she was so, yeah she was in such bad shape. So I wanted to assure myself that she did make it inside, not getting up so something else. Um… I tried to explain that afterwards, because when she is upset like that it’s like, not possible to talk to her at all. Then I tried to explain that it, that’s the way it is. It is my parental responsibility to make sure you’re taken care of when you’re feeling so poorly. So the time after that, I stayed in the car [sigh] and watched as she went inside.  **Interviewer**: Mm. Right. How was that for you? Um, the second time I mean, when you…?  **Participant 7**: Yeah. It, you just have, you just have to [laughter] take it, frankly. It, it’s the way it is. | Participant 7 usually gives the child a ride to BA, but the child does not want her mother to accompany her inside. When the child was ’in bad shape’, the mother felt a parental responsibility to accompany her to the door, controlling and making sure that she is indeed admitted and does not deviate from there. This enraged her child, according to the parent, and the mother tried to explain her motives afterwards, as she feels it is impossible to reach the child when she is upset. After that event, the parent has not tried to accompany her child to the door again, rather waiting in the car until she sees her child go inside. The parent’s comment that ’you just have to take it’ indicates that she feels subdued, without her own autonomy*.* | This parent’s lived space is, in some ways, dominated by the conditions of the BAs, necessitating her to drive to the psychiatric unit yet also restricting her access to that space.  The parent’s lived relation with her daughter is a struggle for control, where the mother doesn’t quite trust her child and tries to stay very close to her, surveilling her, while the teenager is trying to keep her mother at a distance, to break free and gain independence.  Rather than imposing her parental mandate, prepared to face the reactions for the sake of the child’s safety, or letting it go in acceptance, recognizing that her child needs space, there is an undertone of hopelessness, helplessness, and resignation for the parent. While the child is gaining autonomy during BA, the parent is losing her autonomy. |
| **Participant 17**: I, uh, guess I feel that we, we are not very positive to [BA], my husband and I. [sceptical tone, sigh-laughter] Uhm, it sounded, it sounded positive and good but what we are missing in it was uhh, support once you are there. All, all of the focus was directed at him just like resting and not talking to anyone and not… You, you are like there but you are not included. […] You think, you compare with being admitted [conventionally] there and it- you get like support from a psychologist, support from a healthcare counsellor, and the doctor comes to check on you and such, and suddenly you are there yet not there. Um, it feels a bit like a no-man’s-land. **Interviewer**: That you are somewhat invisible when you are there? **Participant 17**: Yes, you are. Your thoughts, reflections, tears and anger are like, not really taken seriously because it’s BA. | The parent experiences a lack of support during the BAs. She speaks in terms of the parents lacking support and speaks in the general form as if she is also the patient, but what she appears to intend is support for her child. The parent expresses disappointment and scepsis about her child not receiving any treatment sessions. She describes it as the child being there yet not there, invisible and out of mind for the staff, as if the child’s thoughts and emotions are taken less seriously when he is admitted with BA. | This parent’s lived relation with the healthcare professionals is one marked by lack.  She relates so strongly to (what she imagines to be) her child’s experience, that she speaks as if from his voice, from the child’s perspective – yet her upset also seems to stem from the parental perspective of ‘my child is being neglected’.  This parent appears disillusioned; she was expecting more from the BAs than what she experiences when her son actually uses these admissions. It seems like a betrayal on behalf of the staff. |

*This table provides examples of the researchers’ work with ‘parts’ in the phenomenological psychological analysis, up to the final stage of returning to the whole.
